# Comparison of Schmallenberg virus antibody levels detected in milk and serum from individual cows

**DOI:** 10.1186/s12917-015-0365-1

**Published:** 2015-03-11

**Authors:** Janet M Daly, Barnabas King, Rachael A Tarlinton, Kevin C Gough, Ben C Maddison, Roger Blowey

**Affiliations:** School of Veterinary Medicine and Science, University of Nottingham, Sutton Bonington, Nottingham, Leicestershire LE12 5RD UK; ADAS Biotechnology Group, School of Veterinary Medicine and Science, University of Nottingham, Sutton Bonington, Nottingham, Leicestershire LE12 5RD UK; Wood Veterinary Group, 125 Bristol Road, Quedgeley, Gloucester, GL2 4NB UK

**Keywords:** Schmallenberg virus, ELISA, Milk, Serum, Antibody

## Abstract

**Background:**

Schmallenberg virus (SBV) is a recently emerged virus of ruminants in Europe. Enzyme-linked immunosorbent assays (ELISA) are commonly used to detect SBV-specific antibodies in bulk tank milk samples to monitor herd exposure to infection. However, it has previously been shown that a bulk tank milk sample can test positive even though the majority of cows within the herd are seronegative for SBV antibodies. Development of a pen-side test to detect antibodies in individual milk samples would potentially provide a cheaper test (for which samples are obtained non-invasively) than testing individual serum samples by ELISA. Therefore, the aim of this study was to investigate the agreement between antibody levels measured in milk and serum.

**Results:**

Corresponding milk and serum samples from 88 cows in two dairy herds in the UK were tested for presence of immunoglobulin G antibodies to SBV using a commercially-available indirect ELISA. A serum neutralisation test (NT) was also performed as a gold standard assay. The ELISA values obtained for the bulk tank milk samples corresponded with the mean values for individual milk samples from each herd (bulk tank milk values were 58% and 73% and mean individual milk values 50% and 63% for herds A and B, respectively). Of the 88 serum samples tested in the NT, 82 (93%) were positive. Although at higher antibody levels, the ELISA values tended to be higher for the individual milk samples than for the corresponding serum samples, the positive predictive value for milk samples was 98% and for serum samples 94%. The serum ELISA was more likely to give false positive results around the lower cut-off value of the assay.

**Conclusions:**

The results indicate that testing of individual milk samples for antibodies against SBV by ELISA could be used to inform decisions in the management of dairy herds such as which, if any, animals to vaccinate.

## Background

Schmallenberg virus (SBV), which emerged recently in Europe, causes subclinical or mild disease in adult ruminants with clinical signs including diarrhoea, fever and drop in milk yield in dairy cattle. However, infection of pregnant animals during a critical period of pregnancy can cause fetal deformities and may result in loss of the fetus or unviable offspring [1]. The first indirect enzyme-linked immunosorbent assay (ELISA) to detect SBV-specific antibodies in serum or milk samples became commercially available shortly after the emergence of SBV [2]. Testing of bulk tank milk samples by ELISA has been advocated as a convenient way to determine herd-level exposure to SBV [3]. With the availability of vaccines against SBV, it has become important to know the value of test results for informing herd management decisions; for example, whether a positive bulk tank milk sample result means that herd-level vaccination is not necessary as natural immunity is present.

Since its emergence, SBV has spread rapidly across Europe and high levels of seroprevalence in cattle have been reported (reviewed in [4]). However, studies have also demonstrated that within-herd seroprevalence is variable. In addition to regional variation in seroprevalence, higher rates have been reported for herds that graze outdoors compared to herds that are housed indoors [5]. Furthermore, in one study, a bulk tank milk sample tested positive although only 25% of serum samples from individual animals within the herd were positive for antibodies to SBV [6].

The aim of this study was to examine the relationship between antibody levels detected in bulk tank milk and individual milk and serum samples from SBV-exposed cows in two herds using a commercially-available ELISA, with a serum neutralisation test as a reference.

## Methods

Blood and milk samples were collected from Holstein-Friesian dairy cows in two herds (49 samples from herd A and 39 from herd B) on 2^nd^ October 2013. A bulk tank milk sample was also obtained from each herd. None of the cows had been vaccinated against SBV. All were clinically healthy at the time of sampling, but clinical signs suggestive of SBV infection (diarrhoea and drop in milk yield) had been observed around one month prior to sampling in herd B. All samples were stored at -20°C until tested. The study was approved by the School of Veterinary Medicine and Science’s Ethical Review Committee.

The presence of immunoglobulin G antibodies to SBV in milk and serum samples was determined using a commercially available indirect ELISA (SVANOVIR® SBV-Ab, Svanova) according to the manufacturer’s instructions. As per the manufacturer’s instructions, the percent positivity (PP) relative to the positive control serum supplied was calculated with a PP of ≥10% considered positive for serum samples and ≥8% for milk samples. Neutralization tests (NT) were performed on serum samples as previously described [7] using SBV strain BH80/11-4 (kindly provided by M. Beer, Friedrich-Loeffler Institute) with the minor modification that cells were fixed in 100% ethanol for 30 minutes then stained for 30 minutes with 0.1% v/v methylene blue in water. The cut-off value for a positive result was set at a titre of 1:8. Milk samples could only be tested by ELISA as milk is toxic to the cells used in the NT.

Positive predictive (the probability that the disease is present when the test is positive) and negative predictive (the probability that the disease is absent when the test is negative) values were calculated for the ELISA using the serum NT as a reference. ELISA results with milk or serum were classified as true positive (TP) or true negative (TN) if in agreement with the serum NT. If results differed from the serum NT, they were classified as false positive (FP) or false negative (FN). Positive predictive value was calculated as TP/(TP + FP) and negative predictive value as TN/(TN + FN) and expressed as a percentage.

Two-sample and paired t-tests as appropriate (with statistical significance set at p < 0.05) were performed using Minitab version 16. Bland-Altman analysis (to evaluate the variability between the serum and milk antibody levels measured by ELISA over the full range of results) was performed using GraphPad Prism v6.

## Results and discussion

The bulk tank milk sample from herd A had an antibody level of 58% and from herd B 73%. Although the ELISA is only semi-quantitative, the mean of the individual milk sample values was consistent with the bulk tank milk sample values. A significantly lower (two-sample t-test, p = 0.037) mean antibody level was obtained for individual milk samples from herd A (50%) than for herd B (63%). Similarly, in a larger published study of bovine viral diarrhoea virus in which milk samples were tested for antibodies, individual milk and bulk tank milk results correlated well [8]. Thus, bulk tank milk testing might indicate the presence of individuals within a herd with lower antibody levels (and therefore at potentially greater risk of infection), but provides no information as to which (or how many) individuals are at potential risk of infection.

In the analysis of samples from individual animals, six cows tested negative in the serum NT (four from herd A and two from herd B). Milk and serum samples from one of these cows also tested negative by ELISA. The other five animals all tested positive by serum ELISA (Table 1A) whereas only two of them tested positive by milk ELISA (Table 1B). Thus, the positive predictive values were 98% and 94% for the milk and serum ELISA, respectively and the negative predictive value for the milk ELISA was 100% but for the serum ELISA was 50%. Thus the serum ELISA was more likely to give both false positive and false negative results. The values obtained in the serum ELISA for the five ‘false positive’ samples were all at or just above the lower cut-off value of 10% (10–11%). Both the positive and the negative predictive values will be influenced by the high prevalence of SBV antibodies in the animals tested; in a high prevalence setting such as this, it is more likely that animals that test positive truly have antibodies to SBV and, conversely, that the negative predictive value is decreased [9].

**Table 1:** **Results of 88 bovine milk and serum samples analysed with a commercial indirect enzyme-linked immunosorbent assay (milk or serum ELISA) or a serum neutralization test (serum NT) for detection of antibodies to Schmallenberg virus**

**(A)**				
		**Serum NT**		**Total**
		**Positive**	**Negative**	
Serum ELISA	Positive	81	5	86
	Negative	1	1	2
	Total	82	6	88
**(B)**				
		**Serum NT**		**Total**
		**Positive**	**Negative**	
Milk ELISA	Positive	82	2	84
	Negative	0	4	4
	Total	82	6	88
**(C)**				
		**Milk ELISA**		**Total**
		**Positive**	**Negative**	
Serum ELISA	Positive	83	3	86
	Negative	1	1	2
	Total	84	4	88

Comparison of (A) serum NT and serum ELISA; (B) serum NT and milk ELISA; (C) serum ELISA and milk ELISA.

**Figure 1:**
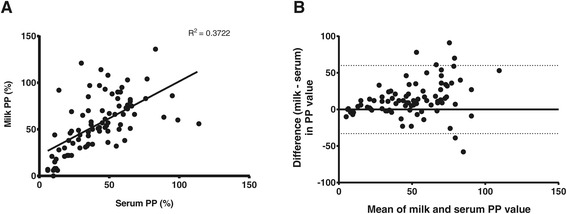
**Antibody levels of 88 paired bovine serum and milk samples analysed using a commercial indirect enzyme-linked immunosorbent assay for antibodies to Schmallenberg virus. (A)** Distribution of observed percent positivity (PP) values. **(B)** Bland-Altman plot of the differences between the milk and serum results against the average PP values for the paired milk and serum samples with 95% limits of agreement shown as dotted lines.

The antibody levels measured in milk samples were significantly (paired t-test, p < 0.001) higher (mean PP 55%, standard error of the mean, SEM 3.13) than in serum samples (mean PP 42%, SEM 2.41). This is in contrast to other studies comparing antibody levels against bovine coronavirus and/or bovine respiratory syncytial virus in matched serum and milk samples, which found good agreement but generally lower antibody levels in milk compared to serum samples [10]. The distribution of the measured PP values is shown in Figure 1A. Bland-Altman analysis revealed a bias of -13.48. Differences between the milk and serum ELISA results were more apparent at mean PP values for the two tests of greater than 50% (Figure 1B).

Protective antibody levels have not been defined for SBV and the indirect ELISA is at best only semi-quantitative. Therefore, if individual testing were conducted in order to inform management decisions such as whether to vaccinate potentially susceptible animals, the cut-off for deciding to vaccinate would be a discretionary one. A negative result in the serum or milk ELISA would clearly indicate a susceptible individual. However, PP values near the assay cut-off should be treated with caution, particularly for the serum ELISA.

As testing a bulk tank milk sample may not provide an accurate reflection of the proportion of a herd that has antibodies, and milk samples can be obtained non-invasively, individual testing of a number of animals can provide an indication of the need to vaccinate the whole herd. However, testing using the currently available indirect ELISAs would, in most cases, be prohibitively expensive. Therefore, if informed decisions are to be made whether or not to vaccinate a dairy herd, a cheaper alternative pen-side test to detect antibodies in individual milk and/or serum samples is required.

## Conclusions

The results from this study suggest that testing of either serum or milk samples from individuals rather than bulk tank milk testing is necessary to identify whether animals within a dairy herd are potentially susceptible to SBV infection.

## Abbreviations

ELISA: Enzyme-linked immunosorbent assay
FN: False negative
FP: False positive
NT: Neutralisation test
PP: Percent positivity
TN: True negative
TP: True positive
SBV: Schmallenberg virus
SEM: Standard error of the mean
